# Mycetoma due to *Nocardia farcinica*

**DOI:** 10.4103/0974-777X.62868

**Published:** 2010

**Authors:** Luna Adhikari, Subhajeet Dey, Ranabir Pal

**Affiliations:** *Department of Microbiology, Sikkim Manipal Institute of Medical Sciences (SMIMS) and Central Referral Hospital (CRH), 5^th^ Mile, Tadong, Gangtok, Sikkim-737 102, India*; 1 *Department of Surgery, Sikkim Manipal Institute of Medical Sciences (SMIMS) and Central Referral Hospital (CRH), 5^th^ Mile, Tadong, Gangtok, Sikkim-737 102, India*; 2 *Department of Community Medicine, Sikkim Manipal Institute of Medical Sciences (SMIMS) and Central Referral Hospital (CRH), 5^th^ Mile, Tadong, Gangtok, Sikkim-737 102, India*

Sir,

Mycetoma is a tropical superficial chronic progressive granulomatous fungal infection of the skin and subcutaneous tissue with sporadic occurrence in India.[1]

A 60-year-old female presented an infection due to *Nocardia farcinica* and demonstrated successful intervention with surgical exploration and prolonged combined chemotherapy. She was brought with a history of multiple discharging sinuses over the right foot and swelling of the same foot since two months after a thorn prick. Physical examination showed chronic skin changes on the leg, with swelling and yellow fluid-draining sinuses. She was admitted in the surgery in-patients, and under strict aseptic conditions, pus and two swabs were collected from the discharging sinuses.

**Figure 1:**
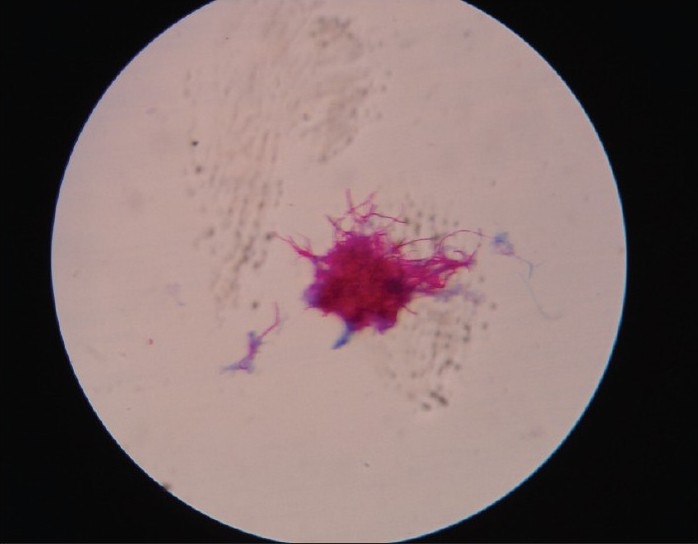
ZN staining of granules

**Figure 2:**
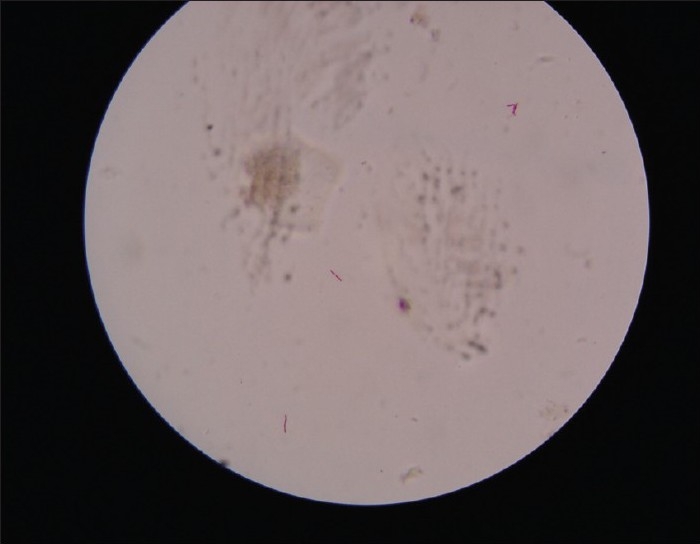
ZN staining of swab showing branching of *Nocardia* spp

**Figure 3:**
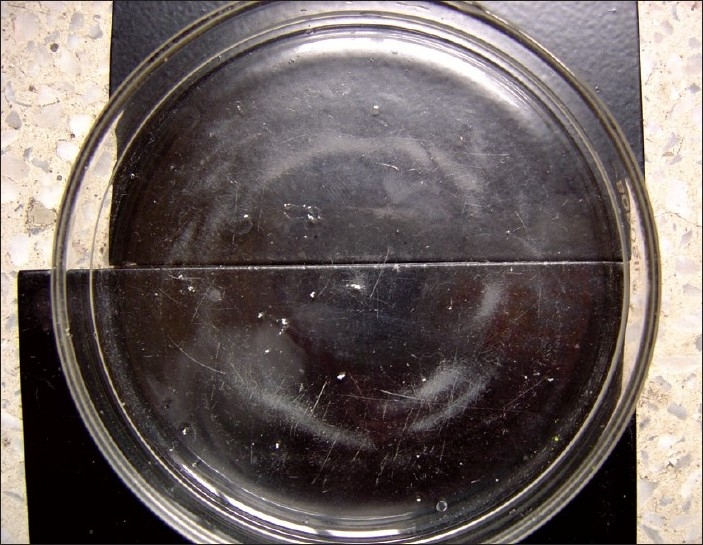
Sterile plate showing granules of *Nocardia* spp as white particle against the dark background

**Figure 4:**
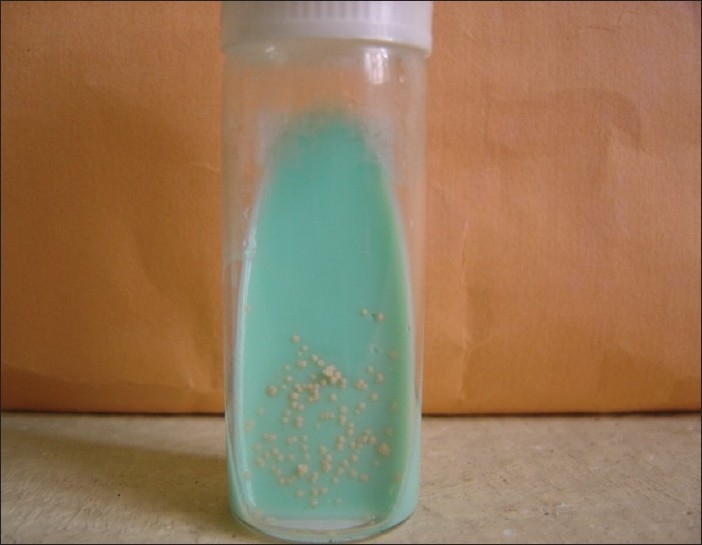
LJ medium showing growth of *Nocardia* spp

Gram's staining of granules revealed Gram positive filamentous bacilli with branching and segmented fragmentation. Modified Ziehl-Neelsen's staining of granules using 1% sulphuric acid revealed acid fast filamentous bacilli. [Figures 1 and 2] Small, soft, irregular white granules were collected from the pus. Sterile plate showing granules as white particle against the dark background [Figure 3] on saline wet mount revealed multilobulated granules with sun ray appearance. [Figure 3] The granules were inoculated on blood agar, Lowenstein-Jensen medium and Sabouraud's dextrous agar slants (with or without chloramphenicol) and incubated at 25°C and 37°C and observed daily for growth. Small, white, wrinkled, heaped colonies appeared after 72 hours of incubation on Lowenstein-Jensen medium [Figure 4] and Sabourauds dextrose agar, where as blood agar got contaminated. Light pink pigmentation appeared after 10 days of incubation. The isolate was identified as *N. farcinica* by its characteristic growth, Gram reaction, acid fastness on modified Ziehl-Neelsen staining using 1% sulphuric acid, positive urease test, positive nitrate reduction test and negative degration of casein (skim milk medium) and positive growth at 45°C for 3 days.[2] The isolate was sensitive to streptomycin, co-trimoxazole, rifampicin. Histopathological examination of the tissue biopsy revealed amorphous aggregates of eosinophilic granules of large clusters of microorganisms resembling fungal hyphae and bacteria, in a background of chronic suppurative inflammation of mixed inflammatory infiltrate cells and stained positively by PAS and Gomori's methenamine silver stain.

Conservative exploration was carried out on the patient after proper written informed consent. She was initially treated with co-trimoxazole and streptomycin and later with dapsone, rifampicin and streptomycin. The patient improved after three months of chemotherapy, the swelling diminished and grain extrusion has ceased. The patient has been able to resume ambulation with normal footwear and recovered uneventfully. In our study, we reported a case of mycetoma due to *N. farcinica*. The actinomycotic mycetoma varies species wise from country to country and place to place. *Nocardia brasiliensis* mycetoma is more commonly reported from different countries like Africa, South America, Mexico, and other parts of India.[3,4] Maiti *et al*. reported from Calcutta that pricking was the most common injury associated with eumycetomas. Lesions in exposed area were more common among agricultural workers with a remarkably lower incidence of *Nocardia.*[5]

The mycetoma of the leg in this case could have been diagnosed early and promptly treated with a high index of suspicion.
